# GSPE Protects against Bleomycin-Induced Pulmonary Fibrosis in Mice via Ameliorating Epithelial Apoptosis through Inhibition of Oxidative Stress

**DOI:** 10.1155/2022/8200189

**Published:** 2022-03-20

**Authors:** Ok Joo Sul, Jin Hyoung Kim, Taehoon Lee, Kwang Won Seo, Hee Jeong Cha, Byungsuk Kwon, Jong-Joon Ahn, You Sook Cho, Yeon-Mok Oh, Yangjin Jegal, Seung Won Ra

**Affiliations:** ^1^Biomedical Research Center, Ulsan University Hospital, School of Medicine, University of Ulsan, Ulsan 44033, Republic of Korea; ^2^Department of Pulmonary and Critical Care Medicine, Ulsan University Hospital, University of Ulsan College of Medicine, Ulsan 44033, Republic of Korea; ^3^Department of Pathology, Ulsan University Hospital, University of Ulsan College of Medicine, Ulsan 44033, Republic of Korea; ^4^School of Biological Science, University of Ulsan, Ulsan 44610, Republic of Korea; ^5^Department Allergy and Clinical Immunology, Asan Medical Center, University of Ulsan College of Medicine, Seoul 05505, Republic of Korea; ^6^Department of Pulmonary and Critical Care Medicine, Asan Medical Center, University of Ulsan College of Medicine, Seoul 05505, Republic of Korea

## Abstract

Idiopathic pulmonary fibrosis (IPF) is a chronic, progressive interstitial lung disease of unknown cause which leads to alveolar epithelial cell apoptosis followed by basement membrane disruption and accumulation of extracellular matrix, destroying the lung architecture. Oxidative stress is involved in the development of alveolar injury, inflammation, and fibrosis. Oxidative stress-mediated alveolar epithelial cell (AEC) apoptosis is suggested to be a key process in the pathogenesis of IPF. Therefore, the present study investigated whether grape seed proanthocyanidin extract (GSPE) could inhibit the development of pulmonary fibrosis via ameliorating epithelial apoptosis through the inhibition of oxidative stress. We found that GSPE significantly ameliorated the histological changes and the level of collagen deposition in bleomycin (BLM)-induced lungs. Moreover, GSPE attenuated lung inflammation by reducing the total number of cells in bronchoalveolar lavage (BAL) fluid and decreasing the expression of IL-6. We observed that the levels of H_2_O_2_ leading to oxidative stress were increased following BLM instillation, which significantly decreased with GSPE treatment both *in vivo* and *in vitro*. These findings showed that GSPE attenuated BLM-induced epithelial apoptosis in the mouse lung and A549 alveolar epithelial cell through the inhibition of oxidative stress. Furthermore, GSPE could attenuate mitochondrial-associated cell apoptosis via decreasing the Bax/Bcl-2 ratio. The present study demonstrates that GSPE could ameliorate bleomycin-induced pulmonary fibrosis in mice via inhibition of epithelial apoptosis through the inhibition of oxidative stress.

## 1. Introduction

Idiopathic pulmonary fibrosis (IPF) is a chronic, progressive interstitial lung disease of unknown cause and is characterized by scarring of interstitial lung tissue and decline in lung function, leading eventually to respiratory failure. The prevalence of IPF is estimated at 13 to 20 cases per 100,000 people per year with a median survival of 3 to 5 years after diagnosis. The prognosis of IPF is significantly worse than in many interstitial lung diseases, and no curable treatments for IPF exist.

Studies have reported the association between genetic, infectious, and environmental factors, such as smoking and pollutants, gastroesophageal reflux disease and aging, and the development of IPF. The mechanism by which these factors influence the pathogenesis of IPF is not fully known. Previously, it has been proposed that IPF is an inflammatory response that eventually leads to chronic lung injury with fibrosis. However, it has recently been suggested that progressive fibrosis involves damage to alveolar epithelial cells (AECs), activation of fibroblasts followed by abnormal wound recovery that leads to fibrosis. Alveolar epithelial damage is the characteristic feature of IPF, in which AEC injury is a significant initial event [1–3].

The production of reactive oxygen species (ROS) is increased under conditions such as exposure to air pollutants and cigarette smoke. ROS is generated by activated macrophages and neutrophils during acute inflammatory reactions and plays an important role as an antibacterial and anticancer. However, continuous ROS exposure may lead to cell damage through the oxidation of DNA, RNA, carbohydrates, proteins, and lipids. There have been many studies showing that an imbalance between oxidants and antioxidants participates in the pathophysiology of IPF and that ROS causes damage to AECs, resulting ultimately in apoptosis. Daniil et al. showed that serum oxidative stress was significantly increased in patients with IPF compared with healthy controls [4], and there are significant correlations between the levels of systemic oxidative stress and disease severity in IPF and the levels of dyspnea.

N-Acetylcysteine, an antioxidant drug, was reported to decrease inflammation and collagen deposition in a mouse model of bleomycin- (BLM-) induced lung fibrosis [5]. However, beneficial effects of NAC on IPF patients have not been demonstrated [6]. The different doses of NAC, the length of time following treatment, or samples from different ethnic origins can be the causes of different results. Oldham et al. recently reported that the efficacy of NAC on IPF patients may be associated with genotype characteristics, suggesting that NAC had a beneficial effect in a subgroup of IPF patients [7].

Grape seed proanthocyanidin extract (GSPE), a flavonoid compound extracted from grape seeds, consists of catechin and epicatechin that form oligomers or polymers. GSPE has various pharmacological properties such as anticarcinogenic [8], antiallergic [9], anti-inflammatory [10], antihypertensive [11], and antiviral activities [12]. Furthermore, GSPE is revealed to be a more effective antioxidant than ascorbic acid and *α*-tocopherol *in vitro* and *in vivo* [13]. Recent studies on GSPE have also reported that GSPE prevents amiodarone-induced lung toxicity [14].

BLM is a chemotherapeutic antibiotic that is used as an anticancer agent but is identified as a profibrotic agent due to it inducing pulmonary fibrosis as a side effect. The model of BLM-induced lung fibrosis is extensively used as an animal model for studying the mechanisms of IPF, as it is easy to perform and is reproducible. BLM causes pulmonary injury, inflammation, and subsequent fibrosis, and ROS generated in response to BLM is involved in the mechanism driving fibrosis in this model [15].

We investigated whether GSPE attenuates BLM-induced ROS production and inflammation levels, resulting in alleviation of epithelial cell apoptosis and eventually fibrosis.

## 2. Materials and Methods

### 2.1. Animal Experiments

Specific pathogen-free (SPF) 8-week-old female C57BL/6 mice, weighing between 18 and 20 g, were purchased from Orient Bio Inc. (Daejeon, Korea). The mice were housed in SPF animal facility and used after 1 week of acclimatization. Experiments were approved by the Institutional Animal Care and Use Committee (IACUC) of Ulsan University.

After one week of acclimation, the mice were randomly divided into five groups (*n* = 6 per group) as follows: after 24 h of inoculation, mice were exposed for 6 days as follows: Experiment #1, (1) PBS (IT)+PBS (IP) group: mice treated with normal saline; (2) BLM (IT)+PBS (IP) group: mice injected with 2 mg/kg of BLM (IT) and PBS (IP); (3) PBS (IT)+GSPE(IP) group: mice injected with PBS (IT) and 90 mg/kg of GSPE; (4–6) BLM (IT)+GSPE (IP, 30, 60, and 90 mg/kg) group: mice treated with 2 mg/kg of BLM and 30, 60, and 90 mg/kg of GSPE; Experiment #2, (1) PBS (IT)+PBS (PO) group: mice treated with normal saline; (2) BLM (IT)+PBS (PO) group: mice injected with 2 mg/kg of BLM and PBS (PO); (3) PBS (IT)+GSPE (PO) group: mice injected with PBS (IT) and 100 mg/kg of GSPE; (4–6) BLM (IT)+GSPE (PO, 50, 100, and 150 mg/kg) group: mice treated with 2 mg/kg of BLM and 50, 100, and 150 mg/kg of GSPE.

### 2.2. Reagents and Antibodies

TNF-*α*, interleukin-1 *β* (IL-1*β*), and IL-6 enzyme-linked immunosorbent assay (ELISA) kits were purchased from BD Biosciences (Cambridge, MA, USA). BLM was purchased from Nippon Kayaku Co. (Ltd., Tokyo, Japan), and GSPE was obtained from SANOFI Co. (Paris, France). Antibody against cPARP (ab32064) was purchased from Abcam (Cambridge, MA, USA). Antibodies against cytokeratin pan (MA5-12231) were obtained from Invitrogen (San Diego, CA, USA). Secondary antibodies tagged with Alexafluor 594 (ab150116) and with Alexafluor 488 (ab150077) were purchased from Abcam. Antibodies against PARP (9532), cleaved caspase-3 (9661), cytochrome c (11940), Bcl-2 (3498), Bax (2772), and Bak (12105) were obtained from Santa Cruz Biotechnology (Santa Cruz, CA, USA). *β*-Actin (A5441) was purchased from Invitrogen and used as internal controls. Horseradish peroxidase- (HRP-) labeled secondary anti-mouse (7076) and anti-rabbit (7074) antibodies were purchased from Cell Signaling Technology (Danvers, MA, USA). CM-H_2_DCFDA and MitoSOX Red were obtained from Invitrogen (San Diego, CA, USA). FITC Annexin V Apoptosis Detection Kit I was purchased from BD Biosciences (San Jose, CA, USA). Hoechst 33342 and MTT (3-(4, 5-dimethylthiazol-2-yl)-2, 5-diphenyltetrazolium bromide) were obtained from Sigma Chemical (St. Louis, MO, USA). M-MLV reverse transcriptase was obtained from Promega (Madison, WI, USA). SYBR Green Real-Time PCR Master Mix was obtained from Enzynomics (Daejeon, South Korea). QIAzol reagent was purchased from Qiagen (Hilden, Germany).

### 2.3. Induction of Lung Injury by BLM

Mice were administered a single dose of 2 mg/kg BLM (Nippon Kayaku Co., Ltd., Tokyo) or saline intratracheally. Intraperitoneal (IP) or per oral (PO) administration of GSPE was performed daily starting at day -4 before BLM (day 0) and lasting 5 times weekly for 3 weeks. All mice were sacrificed 21 days after BLM administration.

### 2.4. Histological Analysis and Ashcroft Scoring

Lung tissues were fixed in 4% paraformaldehyde and dehydrated with graded ethanol. After embedding in paraffin, the sections (4 *μ*m) were prepared and stained with hematoxylin and eosin (H&E) and Masson's trichrome (MT). Lung fibrosis was calculated by semiquantitative analysis of H&E and MT staining using the Ashcroft scoring system as previously described [16]. To quantify the severity of interstitial fibrosis in lung histopathology, each successive field was given a score ranging from 0 (normal lung) to 8 (total fibrous obliteration of the field).

### 2.5. Hydroxyproline Assay

The procedure for quantitation of lung hydroxyproline was performed as previously described [17]. Briefly, whole lung tissue was washed in PBS and hydrolyzed for 18 h in 6 N HCl at 110°C. The pH was adjusted to 6.0 with NaOH titration. The samples were centrifuged, and the pellets were oxidized with chloramine T for 30 minutes. p-Dimethylaminobenzaldehyde was added to each sample, and the samples were incubated at 65°C for 15 minutes. The absorbance was measured at 560 nm on a spectrophotometer. Lung hyroxyproline was quantitated with a standard curve of purified hydroxyproline (Sigma, St. Louis, MO), and the values were corrected for total lung wet weight.

### 2.6. Bronchoalveolar Lavage and Cell Count

On day 7 after BLM administration, bronchoalveolar lavage fluid (BALF) was collected by washing of the lung with an initial 800 *μ*l of sterile PBS, followed by eight 500 *μ*l PBS washes. The fluid was centrifuged, and the supernatant of the first BAL was used for the determination of cytokines. The cell pellet was resuspended in 1 ml of PBS, and the cell number was counted using a hemocytometer.

### 2.7. Immunohistochemical Analysis

From paraffin-embedded lung tissues, 4 *μ*m thick sections were prepared and mounted on glass slides. The sections are deparaffinized with xylene three times each for 15 min and then rehydrated with decreasing alcohol concentrations (100-70%) for 5 min. The protease-induced antigen retrieval was performed, and blocking buffer was added. Next, the slides were incubated overnight at 4°C with anti-PARP p85 fragment polyclonal antibody (Cell Signaling Technology, Danvers, MA, USA), followed by incubation with FITC-conjugated Goat Anti-rabbit IgG secondary antibody (Santa Cruz Biotechnology, Dallas, Texas, USA), and incubated with anti-pan cytokeratin monoclonal antibody PE (Santa Cruz Biotechnology, Dallas, Texas, USA).

### 2.8. RNA Isolation, Purification, and Quantitative Real-Time PCR

Total RNA was extracted from lung tissue samples and cultured A549 cells using TRIZOL reagent. Then, cDNA was synthesized from 1 *μ*g of total RNA using oligo (dT) primers and M-MLV reverse transcriptase. Quantitative PCR was performed using SYBR Green qPCR Master Mix and the appropriate primers on an ABI 7500 Fast Real-Time PCR System (Applied Biosystems, Carlsbad, CA, USA). The housekeeping gene 18S rRNA (RPS) was amplified in parallel with the genes of interest. The following primers were used: Relative expression standardized to RPS was calculated using the comparative cycle threshold method (2^-*ΔΔ*Ct^). The primer sequences used were as follows: 5′-GTGCTCGGCTTCCCGTGCAAC-3′ and 5′-CTCGAA GAGCATGAAGTTGGGC-3′ (GPX); 5′-CTGCCAAGTGATTGGTGCTTCTG-3′ and 5′-AATGGTGCGCTTCGG GTCTGAT-3′ (PRDX); 5′-CAAATGCATGCCGACCTTCCAG-3′ and 5′-GCTGGTTACACTTTTCAGAGCATG-3′ (TRX); 5′-CAAGGAAGCACATGACCGAGCA-3′ and 5′-CTTGTTGCGGTCCATTTCCTC-3′ (RPS).

### 2.9. Measurement of Cytokines

For cytokine measurements, whole lung tissue was homogenized in 10 volumes (*w*/*v*) of ice-cold PBS containing protease inhibitors. Homogenates were centrifuged at 12,000 × *g* at 4°C for 5 min, and the supernatants were collected for analysis. The levels of TNF-*α*, IL-1*β*, and IL-6 were measured by ELISAs according to the manufacturer's instructions (R&D Systems).

### 2.10. Cell Culture

Human alveolar epithelial A549 cells (KCLB, Seoul, Korea) were grown in RPMI 1640 medium (Welgene, Daegu, Korea) supplemented with 10% FBS and 1% penicillin-streptomycin solution at 37°C in a humidified atmosphere with 5% CO_2_. A549 cells were pretreated with GSPE (1 *μ*g/ml) for 4 h and then stimulated with BLM (1 *μ*g/ml) for an additional 40 h.

### 2.11. Western Blot Analysis

Lung tissue samples were homogenized in extraction buffer (50 mM Tris-HCl, pH 8.0, 150 mM NaCl, 1 mM EDTA, 0.5% Nonidet P-40, 0.01% protease inhibitor mixture). After centrifugation for 20 min at 100,000 × *g*, the supernatants were recovered. A549 cells were harvested and treated with the extraction buffer after washing with PBS. Typically, 20 *μ*g of protein per lane was loaded on SDS-PAGE and transferred onto the nitrocellulose membrane. The membranes were blocked for 1 h with 5% skim milk in Tris-buffered saline containing 0.05% Tween 20 (TBST) and were incubated overnight at 4°C with primary antibodies against PARP, cleaved PARP, cleaved caspase-3, cytochrome c, Bax, Bak, Bcl-2, and *β*-actin. After washing with TBST, the membrane was incubated for 1 h with HRP-conjugated secondary antibodies and developed using chemiluminescence HRP substrates.

### 2.12. Measurement of Reactive Oxygen Species (ROS)

Whole lung tissue was homogenized in ice-cold PBS containing protease inhibitors. Homogenates were centrifuged, and the resulting supernatants were used for the assay. H_2_O_2_ levels in homogenates were measured using a hydrogen peroxide assay kit (Cell BioLabs, San Diego, CA) according to the manufacturer's protocol. Briefly, 25 *μ*l of sample or diluted H_2_O_2_ standards was added to 96-well plates, mixed with 25 *μ*l of working solution, and incubated for 30 min at room temperature. Absorbance was monitored at 540 nm, and H_2_O_2_ concentrations were determined using the standard curve.

To measure intracellular ROS, A549 cells were pretreated with GSPE (1 *μ*g/ml) for 4 h and then stimulated with BLM (1 *μ*g/ml) for an additional 24 h. Then, the cells were rinsed briefly in PBS and incubated with culture medium containing 5 *μ*M CM-H_2_DCFDA at 37°C for 30 min in the dark. Fluorescence was detected by flow cytometry (FACSCanto II), and the data were analyzed by FlowJo V10 software (Tree Star Inc., San Carlos, CA).

To perform mitochondrial ROS (mtROS) measurements, A549 cells were pretreated with GSPE (1 *μ*g/ml) for 4 h and then stimulated with BLM (1 *μ*g/ml) for an additional 24 h. After treatment, the cells were stained with MitoSOX Red (5 *μ*M) for 30 min and then washed three times with PBS. mtROS was assessed by using flow cytometry, and the data were analyzed by FlowJo V10.

### 2.13. Cell Viability Assay

Cell viability was determined by MTT assay. A549 cells were seeded in a 96-well plate at 3 × 10^4^ cells per well and were allowed to grow to 80% confluency. The cells were then pretreated with GSPE at various concentrations (0, 0.5, 1, 5, 10, and 20 *μ*g/ml) for 4 h and stimulated with or without BLM at different concentrations (0, 0.5, 1, 10, 20, and 40 *μ*g/ml). Next, the cells were incubated with 0.5 mg/ml of thiazolyl blue tetrazolium bromide for 3 h. The MTT was removed, and 100 *μ*lL of dimethylformamide (DMSO) was added. Then, the optical densities of the samples were read at 540 nm on a spectrophotometer.

### 2.14. Apoptosis Assay

The A549 cells were harvested, washed twice with cold PBS, and resuspended in 100 *μ*l annexin binding buffer. The cells were then stained for 15 min at RT in the dark with 5 *μ*l FITC-conjugated annexin V and 5 *μ*l propidium iodide (PI) using the FITC-Annexin V Apoptosis Detection Kit (BD Bioscience, Heidelberg, Germany), according to the manufacturer's instructions, and analyzed by flow cytometry.

### 2.15. Statistics

Values are expressed as means ± SEM. Each series of experiments was repeated at least three times. Since the sample means are normally distributed, the one-way ANOVA was used for more than two groups' comparison, followed by Bonferroni posttests. The *t*-test method was used for a two-group comparison. The statistical analysis was performed using GraphPad Prism 5 (GraphPad Prism Software Inc., CA, USA) and SPSS 24.0 (SPSS, Inc., IL, USA). A *p* value < 0.05 was considered statistically significant.

## 3. Results

### 3.1. GSPE Attenuated BLM-Induced Lung Fibrosis

**Figure 1:**
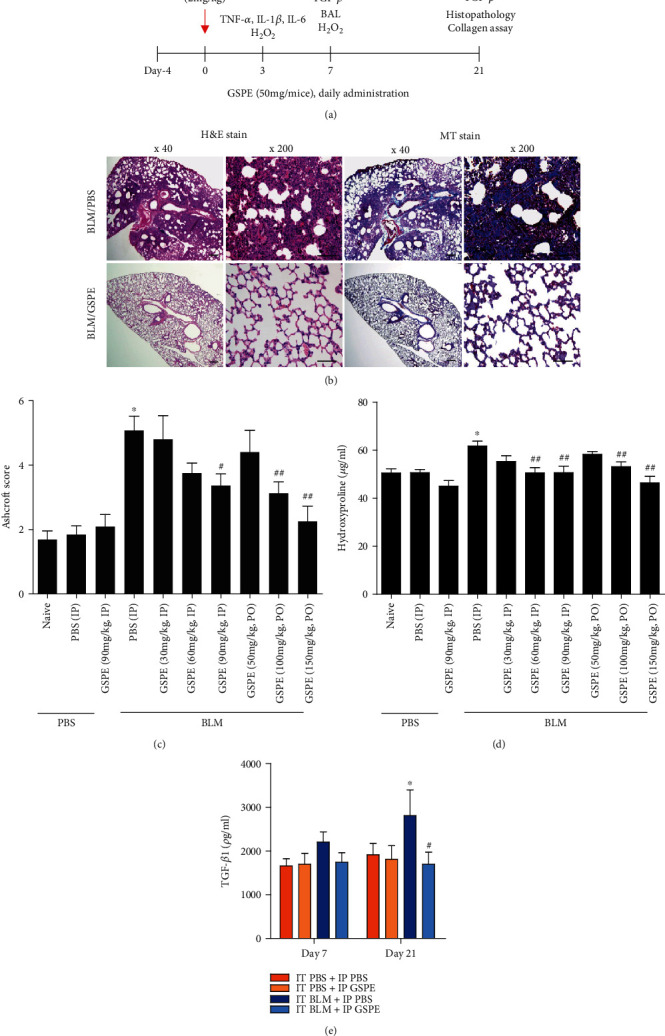
Grape seed proanthocyanidin extract (GSPE) attenuated bleomycin- (BLM-) induced lung fibrosis. (a) Experimental protocol for bleomycin-induced pulmonary fibrosis and timetable for drug administration or sampling. Intraperitoneal (IP) or peroral (PO) administration of GSPE started from day 4 to day 21. Administration of intratracheal (IT) BLM on day 0. Sampling for cytokines, H_2_O_2_ and collagen bioassay, immunohistochemistry (IHC), and bronchoalveolar lavage (BAL) at different time points. (b) Representative photomicrographs of lung pathology in the BLM+PBS group and BLM+GSPE group at 21 days post-BLM. Left shows that lung sections are stained with hematoxylin–eosin. A marked peribronchial interstitial infiltration of inflammatory cells and alveolar edema are present in PBS-treated animals. Treatment with GSPE attenuated these pulmonary lesions. Right shows lung sections stained with Masson trichrome at 21 days post-BLM. BLM caused a significant increase in the interstitial collagen of the lung. The interstitial collagen in the lung was diminished by treatment with GSPE. Original magnifications, ×40 and ×200. Scale bars, 200 *μ*m and 100 *μ*m. (c) Ashcroft scores in experimental groups. Treatment with GSPE reduced the Ashcroft score in a dose-dependent manner. (d) Hydroxyproline levels in experimental groups. Treatment with GSPE reduced lung content of hydroxyproline in a dose-dependent manner. (e) Expression of fibrogenic cytokine, TGF-*β*1. Intratracheal (IT) administration of BLM-induced increased levels of TGF-*β*1 on day 21. Compared with the BLM+PBS treatment group, the level of TGF-*β*1 was significantly lower in the BLM+GSPE treatment group on day 21. Data are expressed as the mean ± SEM (*n* = 4–6 per group) and are representative of three to five independent experiments. ^#^*p* < 0.01 versus the IT PBS+IP PBS group. ^∗^*p* < 0.05 and ^∗∗^*p* < 0.01 versus the IT BLM (bleomycin)+IP PBS group.

To investigate the effect of GSPE on the pathogenesis of lung fibrosis, we established a BLM-induced lung fibrosis mouse model (Figure 1(a)). Mice were treated with GSPE (IP or PO) or saline from -4 days for 25 days before intratracheal administration of BLM to induce lung fibrosis on 0 days. For histopathological analysis, lung sections were stained with hematoxylin and eosin (H&E) and Masson's trichrome (MT). Interestingly, we observed that peribronchial interstitial infiltration with inflammatory cells and alveolar edema was increased in the BLM-treated group (Figures 1(a) and 1(b)). However, treatment with GSPE attenuated these BLM-induced lung lesions (Figure 1(b), left), as shown by the H&E-stained lung sections. Furthermore, histological sections stained with Masson's trichrome showed that increased interstitial collagen was present in the BLM-treated group in Figure 1(d). However, administration of GSPE alleviated the interstitial collagen in the lung induced by BLM (Figure 1(b), right). In addition, the fibrosis scores of lung fibrosis in the BLM-treated group were significantly higher than those in PBS- and GSPE-treated control groups. In contrast, the Ashcroft scores of lung fibrosis were lower in both the BLM+IP GSPE and BLM+PO GSPE groups in a dose-dependent manner compared with the BLM+PBS group (Figure 1(c).

To assess whether GSPE reduced the level of collagen deposition in BLM-induced lung, we next measured the lung hydroxyproline content, a fibrotic marker of deposited collagen on day 21. Consistent with the histopathological results of Masson's trichrome staining, we found that the lung hydroxyproline content was considerably elevated in the BLM group compared with the PBS-treated control mice. However, the BLM+IP GSPE group exhibited a marked reduction of hydroxyproline content compared with the BLM+PBS group (55.3, 51.9, and 50.4 vs. 62.9 *μ*g/ml). (Figure 1(d)). In addition, to determine the effects of GSPE on the expression of fibrogenic cytokines, we measured the expression of TGF-*β* in the lungs at days 7 and 21 after BLM instillation. We observed that the protein levels of TGF-*β* increased at day 21 but that GSPE treatment substantially reduced the fibrogenic cytokine at 21 days (Figure 1(e)). Taken together, these results indicate that GSPE significantly improves lung fibrosis-triggered BLM.

### 3.2. GSPE Attenuates BLM-Induced Lung Inflammation

**Figure 2:**
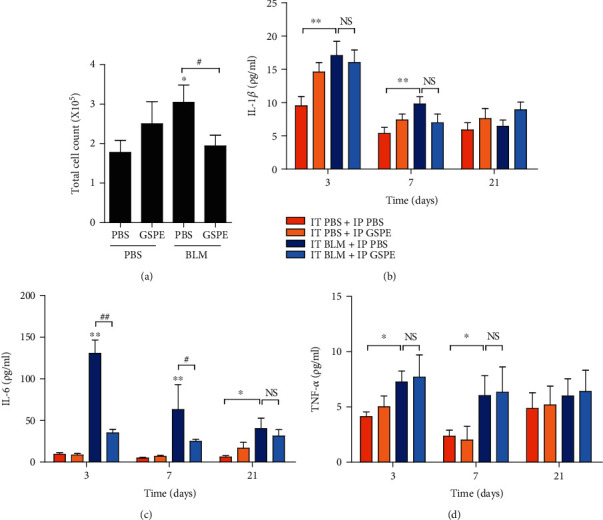
Grape seed proanthocyanidin extract (GSPE) attenuates bleomycin- (BLM-) induced lung inflammation. (a) Total numbers of cells in bronchoalveolar lavage (BAL) fluid were determined at day 7 of BLM instillation to assess the protective effects of GSPE on BLM-induced lung inflammation. Intratracheal (IT) administration of BLM increased total cell counts in BALF on day 7. Compared with the BLM+PBS group, total cell counts in BALF were significantly lower in the BLM+GSPE group on day 7. (b–d) The levels of TNF-*α*, IL-1ß, and IL-6 were measured by ELISA in whole lung tissue homogenates. IT administration of BLM induced increased levels of TNF-*α* and IL-1ß on days 3 and 7 and IL-6 on days 3, 7, and 21. Compared with the BLM+PBS treatment group, the level of IL-6 was significantly lower in the BLM+GSPE treatment group on days 3 and 7. Treatment with GSPE did not reduce the levels of TNF-*α* and IL-1ß. Data are expressed as the mean ± SEM (*n* = 3–5 per group) and are representative of three independent experiments. ^∗∗^*p* < 0.01 and ^∗^*p* < 0.05 versus the IT PBS+IP PBS group. ^##^*p* < 0.01 and ^#^*p* < 0.05 versus the IT BLM+IP PBS group. NS: not significant.

To evaluate the effect of GSPE on initial lung inflammation induced by BLM, we determined the total number of cells in BALF at day 7 of BLM instillation. Consistent with the H&E staining results, the total number of cells in the BLM-treated group was significantly higher than in the PBS-treated control group. However, treatment of BLM-induced mice with 90 mg/kg GSPE dramatically decreased total cell numbers when compared to the BLM+PBS group (Figure 2(a)). As expected, the administration of BLM increased the levels of TNF-*α* and IL-1*β* on days 3 and 7 and IL-6 on days 3, 7, and 21. We next measured whether GSPE reduces the BLM-induced increases in proinflammatory cytokines in the lung tissue. Interestingly, GSPE significantly attenuated the expression of IL-6 on days 3 and 7 (Figure 1(e)) but had no difference in the levels of TNF-*α* and IL-1ß. These data showed that GSPE attenuated lung inflammation via reducing the numbers of total cells, macrophages, neutrophils, and lymphocytes and decreasing the expression of IL-6 (Figure 2(b)).

### 3.3. GSPE Attenuates BLM-Induced Oxidative Damage *In Vivo* and *In Vitro*

**Figure 3:**
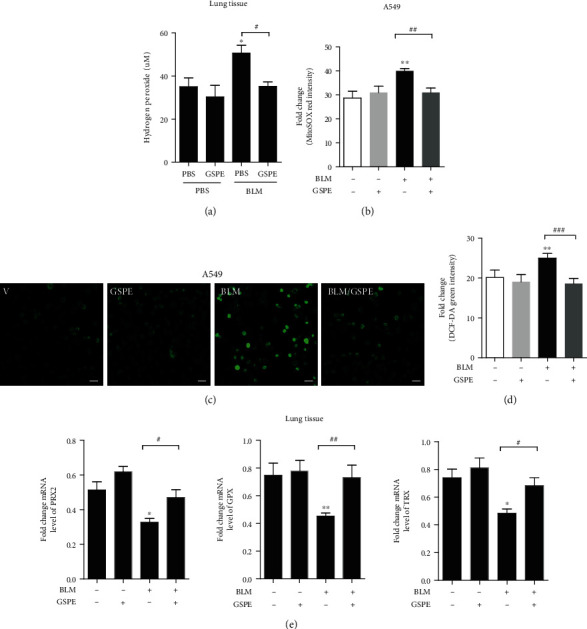
Grape seed proanthocyanidin extract (GSPE) attenuates bleomycin- (BLM-) induced oxidative damage *in vivo* and *in vitro*. (a) The levels of H_2_O_2_ were measured in whole lung tissue homogenates on day 3. Intratracheal (IT) administration of BLM increased levels of H_2_O_2_ on day 3. Compared with the BLM+PBS treatment group, the level of H_2_O_2_ was significantly lower in the BLM+GSPE treatment group on day 3. (b, c) A549 cells were pretreated with GSPE (1 *μ*g/ml) for 4 h and then stimulated with BLM (1 *μ*g/ml) for an additional 24 h. The production of mitochondrial ROS by staining with MitoSOX using flow cytometry was detected. Intracellular ROS were detected by staining with DCF-DA using confocal microscopy (c) and flow cytometry (d). GSPE significantly decreased intracellular ROS and mitochondrial ROS production induced after BLM treatment. (e) mRNA levels of several antioxidant enzymes were measured by qRT-PCR. GSPE treatment restored the mRNA expressions of PRX2, GPX, and TRX reduced by BLM. Scale bar = 20 *μ*m. Data are expressed as the mean ± SEM (*n* = 3–5 per group) and are representative of three independent experiments. ^∗^*p* < 0.05 and ^∗∗^*p* < 0.01 versus the PBS+PBS group or nontreated cells.

Previous studies have shown that oxidative stress plays a major role in BLM-induced lung fibrosis. To evaluate whether GSPE inhibited BLM-induced oxidative damage *in vivo*, we measured ROS generation at day 3 of BLM instillation. The levels of H_2_O_2_ were increased by BLM on day 3 compared to those of the PBS-treated controls. However, the BLM+GSPE group ameliorated the levels of H_2_O_2_ compared to the BLM+vehicle group, suggesting that GSPE considerably prevented BLM-induced oxidative stress (Figure 3(a)). Next, to evaluate whether GSPE inhibited BLM-induced oxidative stress *in vitro*, we measured ROS generation at day 3 of BLM instillation in A549 cells. The cells were pretreated with GSPE (1 *μ*g/ml) for 4 h and then stimulated with BLM (1 *μ*g/ml) for an additional 24 h. The production of mitochondrial ROS was detected by staining with MitoSOX using flow cytometry (Figure 3(b)), and intracellular ROS levels were detected by staining with DCF-DA using confocal microscopy (Figure 3(c)) and flow cytometry (Figure 3(d)). GSPE significantly decreased mitochondrial ROS and intracellular ROS production induced after BLM treatment. Impaired expression of these antioxidants can lead to an overload of ROS. In IPF, changes in the antioxidant defense system are associated with increased oxidant production. Therefore, since ROS levels increasing in response to BLM may be a result of the inhibited expression of antioxidant enzymes, we measured the mRNA level of several antioxidant enzymes. Interestingly, BLM decreased the protein expressions of Peroxiredoxin 2 (PRX2), glutathione peroxidase (GPX), and thioredoxin (TRX) in lung tissues. However, GSPE treatment restored the expressions of these genes reduced by BLM, indicating that these genes contributed to the decreased oxidant burden (Figure 3(c)).

### 3.4. GSPE Attenuates BLM-Induced Apoptosis in the Lung and AECs

**Figure 4:**
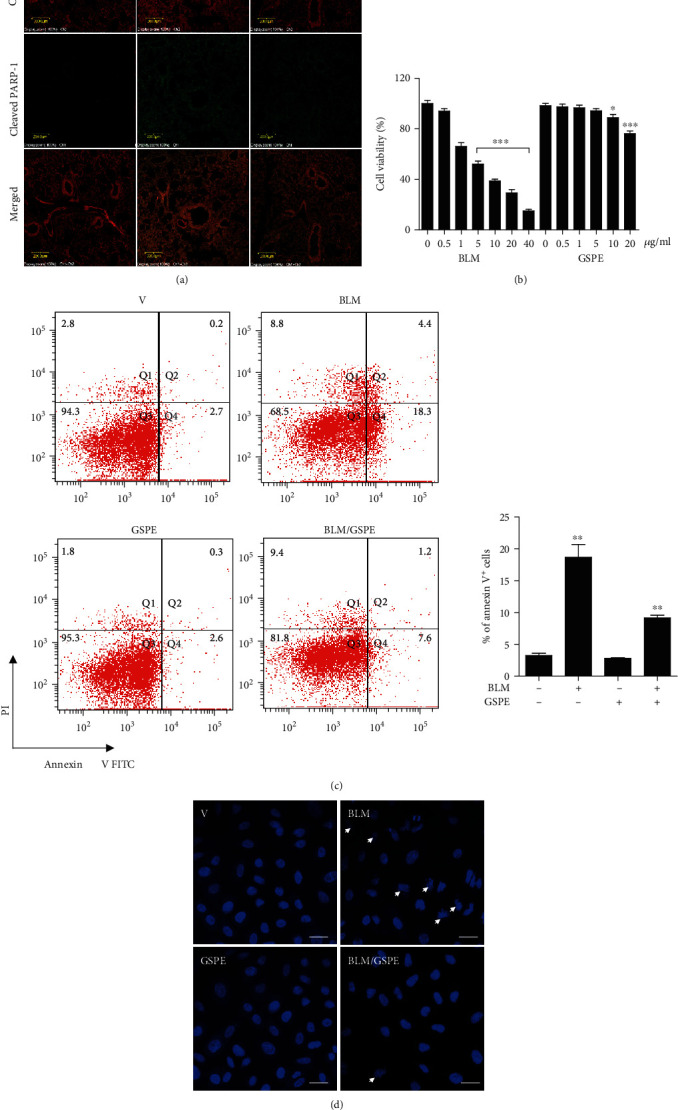
Grape seed proanthocyanidin extract (GSPE) attenuates bleomycin- (BLM-) induced apoptosis in the lung and alveolar epithelial cells. (a) To investigate the effects of GSPE on BLM-induced alveolar epithelial cell apoptosis on day 7, the slides from paraffin-embedded lung tissues were incubated overnight at 4°C with anti-PARP p85 fragment polyclonal antibody, followed by incubation with FITC-conjugated Goat Anti-rabbit IgG secondary antibody, and incubated with anti-pan cytokeratin monoclonal antibody PE. Double staining for cytokeratin (red) and cleaved PARP (green) showed more epithelial apoptosis in the BLM+PBS group than in the BLM+GSPE group. Scale bar = 10 *μ*m. (b) Lung epithelial A549 cells were treated with GSPE (0–20 *μ*g/ml) for 4 h before the addition of BLM (0–40 *μ*g/ml) for 48 h. Cell viability was measured using the MTT assay. At doses of 0.5, 1, and 5 *μ*g/ml, no difference was observed in cell viability after GSPE treatment. In addition, significant decreases in cell viability were observed at concentrations higher than 1 *μ*g/ml after BLM treatment; at 10 *μ*g/ml, cell viability was decreased to 15%. (c) The cells were then stained for 15 min at RT in the dark with 5 *μ*l FITC-conjugated annexin V and 5 *μ*l propidium iodide (PI). GSPE treatment dramatically reduced the fraction of annexin V-positive cells compared with that of BLM administration. (d) Hoechst 33342 staining was performed to analyze apoptosis. Apoptotic cells (indicated by arrows) were reduced in the BLM+GSPE group than those in the BLM+vehicle group. Scale bar, 20 *μ*m. Data are expressed as the mean ± SEM (*n* = 3–5 per group) and are representative of three independent experiments. ^∗∗^*p* < 0.01 and ^∗∗∗^*p* < 0.001 in comparison with nontreated cells.

Alveolar epithelium, which represents 99% of the surface area of the lung, is composed of type I and type II AECs. Apoptosis in the alveolar epithelium (AECs) is essential in the pathogenesis of lung fibrosis [18]. Therefore, we investigated whether GSPE has an antiapoptotic effect on lung epithelial cells. First, we analyzed cleaved PARP*-*1 (cPARP-1) as a marker for detecting apoptotic cells in lung tissues using immunohistochemical staining. Colocalization between cytokeratin and cPARP-1 confirms the apoptotic status of epithelial cells. Interestingly, double staining for cytokeratin (red) and cleaved PARP (green) showed that epithelial apoptosis was not found in the PBS-treated control group but increased in the BLM+vehicle group at day 7 of BLM instillation. However, the cleaved PARP-1 expression increased by BLM administration was significantly decreased by GSPE in the lung (Figure 4(a)). Next, we performed the MTT assay for assessing cell viability in lung epithelial cells. At doses of 0.5, 1, and 5 *μ*g/ml, no significant change in cell viability was observed with GSPE treatment, while at concentrations of 10 to 20 *μ*g/ml, suppression of cell viability was detected. Moreover, the cell viability decreased at concentrations higher than 1 *μ*g/ml after BLM treatment, and at 10 *μ*g/ml, cell viability was decreased to 15% (Figure 4(b)). Additionally, BLM increased an increase of ROS, which leads to oxidative stress and apoptosis in the lung [19]. To identify the effects of GSPE on BLM-induced apoptosis, we performed annexin V-FITC/PI staining. The fraction of annexin V-positive cells decreased dramatically after GSPE treatment compared with that of BLM administration (Figure 4(c)). Next, we performed Hoechst 33342 staining to analyze apoptosis. Positive apoptotic cells show the characteristic fragmented nuclei. As expected, apoptotic cells were reduced in the BLM+GSPE group compared to those in the BLM+vehicle group (Figure 4(d)). These results indicate that GSPE attenuated BLM-induced epithelial apoptosis in the mouse lung and A549 AECs.

### 3.5. GSPE Inhibits BLM-Induced Epithelial Cell Apoptosis via Downregulating of Bax/Bcl-2 Expression

**Figure 5:**
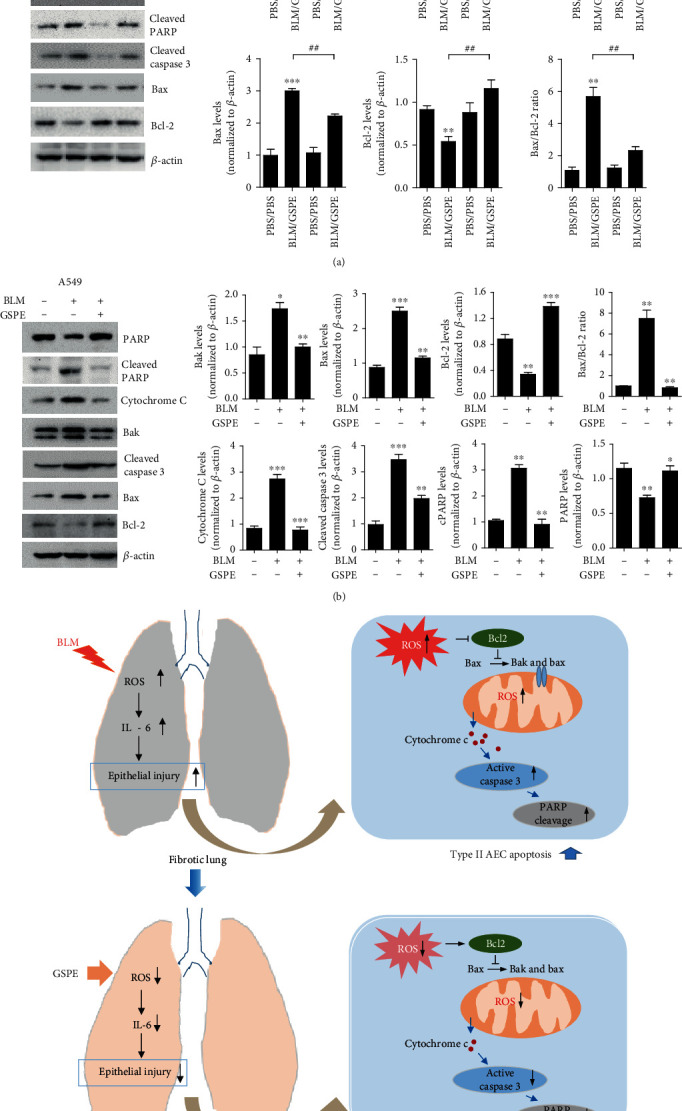
Grape seed proanthocyanidin extract (GSPE) attenuates bleomycin- (BLM-) induced epithelial cell apoptosis via downregulating of Bax/Bcl-2 expression. (a, b) Expressions of the proteins associated with mitochondrial apoptosis after BLM treatment were detected by western blotting analysis in lung tissues and A549 cells. The protein levels of PARP, cPARP, cleaved caspase 3, Bax, and Bcl-2 in lung tissues and PARP, cPARP, cytochrome c, Bak, cleaved caspase 3, Bax, and Bcl-2 in A549 cells were increased after BLM administration. However, expressions of these proteins were reversed by GSPE treatment and the Bax/Bcl-2 ratio was also decreased. (c) Schematic diagram of proposed pathways. GSPE has protective effects in BLM-induced lung fibrosis by reducing oxidative stress and reducing AEC apoptosis in the lung. Data are expressed as the mean ± SEM (*n* = 3-5 per group) and are representative of three independent experiments. ^∗^*p* < 0.05, ^∗∗^*p* < 0.01, and ^∗∗∗^*p* < 0.001 versus the PBS+PBS group or nontreated cells.

Previous studies have reported that BLM functions via mitochondria-dependent apoptotic pathway in response to oxidant injury [20–22]. Therefore, we also measured the expression of these proteins associated with mitochondrial apoptosis in BLM-treated cells. Western blotting analysis showed that BLM induced the protein expression of cytochrome c, cleaved caspase-3, and cleaved PARP in lung tissues. However, GSPE treatment reversed the expression of apoptotic proteins increased by BLM. BLM exposure causes mitochondrial localization of Bax, resulting in the release of cytochrome c [23]. Bax is associated with mitochondrial-dependent apoptosis. Interestingly, BLM induced an increase of Bax and Bak, which were inhibited by GSPE. Moreover, GSPE restored BLM-mediated Bcl-2 and PARP levels (Figure 5(a)). We found that GSPE treatment could also decrease the Bax/Bcl-2 ratio. Next, we used the A549 type II AEC cell line to investigate BLM-induced apoptosis of lung epithelial cells. Expectedly, we observed that proapoptotic proteins including Bax and Bak were increased after BLM treatment, whereas GSPE restored the expression of these proteins. In addition, antiapoptotic protein, Bcl-2, which was decreased by BLM, yet increased after GSPE. We also found that BLM increased the protein levels of cPARP, cytochrome c, and cleaved caspase 3, whereas GSPE suppressed the increase of these proteins induced after BML treatment. PARP expression induced by BLM was reversed by GSPE (Figure 5(b)). Taken together, our findings suggested that GSPE might protect the BLM-mediated apoptosis induced by oxidative stress via downregulating the Bax/Bcl2 ratio.

## 4. Discussion

IPF is characterized by a sequence of events starting with alveolar epithelial microinjuries leading to AEC apoptosis and basement membrane disruption followed by the formation of fibroblastic foci and accumulation of extracellular matrix. This results in the subsequent destruction of the lung architecture. The prognosis of IPF is significantly worse than in many interstitial lung diseases, and no effective treatments for IPF exist. Pirfenidone and nintedanib, as well as corticosteroids and other immunosuppressive agents, are used to treat pulmonary fibrosis. However, these clinical drugs have limited efficacy and some side effects. Therefore, new and effective treatments are urgently needed. GSPE, a potent antioxidant, is more effective than ascorbic acid and vitamin E and has anti-inflammatory effects [13, 24]. Considering the antioxidant effects of GSPE and the crucial role of its in pulmonary fibrosis, we hypothesized that GSPE could inhibit the development of pulmonary fibrosis via ameliorating epithelial apoptosis through the inhibition of oxidative stress. To investigate the effect of GSPE on the pathogenesis of lung fibrosis, we first established a BLM-induced lung fibrosis mouse model (Figure 1(a)). H&E, Masson's trichrome staining, and Ashcroft score assessment showed the inhibitory effect of GSPE on BLM-mediated lung fibrosis in mice (Figures 1(b) and 1(c)). GSPE also reduced the level of collagen deposition in BLM-induced lung (Figure 1(d)). These results show that GSPE exerted protective effects via alleviating pathological changes in a BLM-induced lung fibrosis model of mice.

Several studies have reported that oxidative stress was associated with the development of alveolar injury, inflammation, and fibrosis [25]. Oxidative stress might promote a fibrotic microenvironment, where fibroblasts were resistant to the damaging effects of ROS in IPF, but epithelial cells were relatively more sensitive to oxidative stress [26]. Lung myofibroblasts are known to secrete hydrogen peroxide, leading to mediated fibrogenic effects and inducing epithelial apoptosis [27]. Moreover, neutrophils produce matrix-degrading enzymes such as neutrophil elastase and release an excessive amount of ROS that may induce cell and tissue injury. Modulating oxidative stress might be a powerful way to prevent the progression of lung fibrosis. Interestingly, a previous report showed that sivestat, a neutrophil elastase inhibitor, could ameliorate BLM-induced lung fibrosis [28]. Moreover, deficiency of the antioxidant glutathione (GSH) in epithelial lining fluid can increase cell susceptibility to oxidant-mediated injury, which contributes to the initiation and progression of fibrosis [29]. BLM can cause severe lung fibrosis in both animals and humans [30–34]. BLM is involved in the production of ROS, leading to increased oxidative stress, which results in inflammation and fibrosis [35]. AECs are the main target of oxidative stress-induced lung injury leading to fibrosis [36]. In the present study, we found that BLM instillation increased the levels of ROS leading to oxidative stress in mouse lung tissues. However, GSPE treatment significantly decreased BLM-induced ROS levels. These results indicate that BLM-induced oxidative stress can be ameliorated by treatment with GSPE (Figure 3(a)). *In vitro*, our results showed that intracellular ROS and mitochondrial ROS were significantly increased after BLM treatment, while BLM-induced ROS were alleviated after GSPE treatment (Figure 3(b)). We suggested that these beneficial effects are associated with suppressed production of ROS and inflammation and the prevention of epithelial cell apoptosis in the lungs.

Cytokines and growth factors are involved in both the early inflammatory and late fibrotic phases of lung fibrosis. We examined the effect of GSPE on initial lung inflammation induced by BLM. Administration of BLM induced the levels of TNF-*α* and IL-1ß on days 3 and 7 and IL-6 on days 3, 7, and 21, while GSPE significantly attenuated the expression of IL-6 on days 3 and 7 but had no difference in the levels of TNF-*α* and IL-1ß (Figure 2(b)). IL-6 has been implicated in the pathogenesis of many inflammatory disorders of the lung, including IPF, ARDS, and chronic obstructive pulmonary disease [37, 38]. Saito et al. reported that in IL6-deficient mice, BLM-induced inflammatory cell accumulation in the alveolar space, an increase in TGF-*β*1 and CCL3 expression, and fibrotic changes of the lung after BLM were attenuated [39]. IL-6 is enhanced in mice and humans with pulmonary fibrosis [40, 41]. Several genetic studies revealed the significant association between IL-6 and the development of fibrosis in animals and humans [37, 42]. In lung fibroblasts obtained from patients with IPF, IL-6 seems to promote fibroblast proliferation, which contributes to the development of fibrosis compared with lung fibroblasts obtained from normal patients [43]. In the lung, asbestos-induced oxidative stress increased IL-6 expression, triggering both inflammatory and fibrotic processes, which was significantly suppressed by antioxidants including N-acetylcysteine (NAC) [44]. Additionally, we also observed that GSPE instillation also ameliorated the total number of cells in BALF increased by BLM instillation at day 7 (Figure 2(a)). These data indicated that GSPE exerted protective effects via the downregulation of IL-6 proinflammatory cytokine, as well as by alleviating the number of inflammatory cells in the BAL fluid.

Apoptosis, the process of programmed cell death, plays an important role in normal lung homeostasis and the pathogenesis of various lung diseases including IPF [45]. AEC injury is suggested to be a key process in the pathogenesis of IPF [46, 47]. Repeated injury to the AEC leads to apoptosis, followed by aberrant lung repair and fibroblast activation, resulting in progressive fibrosis. Shulamit et al. reported that initial bleomycin-induced oxidative stress causes a direct apoptosis of lung epithelial cells through caspase activation in a murine lung epithelial (MLE) cell line [19]. In addition, EMT is a pathophysiological process for an epithelial cell to undergo a conversion to a mesenchymal phenotype, which may play a significant role in lung fibrosis. EMT characteristics were detected in bleomycin-treated A549 cells [48]. BLM induced alveolar epithelial cell apoptosis through a mitochondrial-dependent pathway, while other studies showed that Fas/FasL pathway is involved in BLM-induced apoptosis and Fas was also overexpressed in alveolar epithelial cells in lung tissues of human idiopathic pulmonary fibrosis [49–51]. However, Shulamit et al. reported that BLM-induced ROS triggered the initial apoptosis in the fibrotic process and subsequent increase of Fas and FasL level further sensitized cells to Fas-induced apoptosis [19]. Therefore, the Fas/FasL apoptotic pathway appears to take place at a later time and seems essential to clear intra-alveolar granulated tissue [52].

AEC apoptosis is an important early event in BLM-induced lung fibrosis, and in this model, suppression of apoptosis ameliorated lung fibrosis [53, 54]. Thus, new therapies based on the inhibition of apoptosis may prove advantageous for the treatment of patients with IPF. Oxidative stress is known to mediate poly-ADP-ribose polymerase 1 (PARP-1) activation, triggering mitochondrial dysfunction, which leads to caspase-independent cell death [55]. PARP-1 is a major substrate of caspases-3, and its cleavage is a valuable marker of apoptosis [56]. PARP-1 is critical for cellular processes such as DNA repair and transcription. In the present study, double staining for cytokeratin (red) and cleaved PARP (green) showed that in the PBS-treated control group, epithelial apoptosis was not found but increased in the BLM+vehicle group on day 7 after BLM instillation. However, the cleaved PARP-1 expression increased after BLM administration and was significantly decreased by GSPE. Moreover, Figures 4(c) and 4(d) show that GSPE could repress type II AEC apoptosis, as evaluated by morphological alterations of nuclear by Hoechst 33342 staining and by annexin V-positive cells, suggesting that GSPE could attenuate BLM-induced apoptosis via ameliorating epithelial apoptosis.

Caspases are a family of proteolytic enzymes, which play an essential role in AEC apoptosis [57]. It is activated through the intrinsic or extrinsic pathways of apoptotic signaling, ultimately leading to the destruction of the cell. The extrinsic pathway, the death receptor pathway, links death receptors such as Fas and tumor necrosis factor (TNF) receptor 1 and caspase-8 [58], whereas the intrinsic pathway, the mitochondrial pathway, occurs through stimuli such as drugs, infectious agents, and ROS [59, 60]. These stimuli lead to increased expression of proapoptotic proteins including Bax and Bak and reduced expression of antiapoptotic proteins including Bcl-2 and Bcl-xL. With a change in mitochondrial membrane potential, cytochrome c is released from the mitochondria to the cytosol, which induces the formation of the functional apoptosome, resulting in the activation of caspase-9 and a cascade of activation events for the effector caspases-3 [61]. Several studies have shown that BLM induces ROS-induced mitochondrial cell death [20–22]. Moreover, Maeyama et al. found that, in the epithelial cells of IPF patients, the levels of caspase-3 were increased [62]. Consistent with these findings, we also observed an increase in the protein level of caspase-3 in mouse lung homogenates after BLM instillation, whereas GSPE suppressed BLM-induced caspase-3 levels. Plataki et al. also detected Bax induction and Bcl-2 reduction in epithelial cells from IPF patients [63]. Additionally, Bax protein was increased in epithelial cells, whereas the expression level of Bcl-2 protein showed no difference in a study of BLM-induced pulmonary fibrosis in mice [64]. In this study, we also showed that BLM induced the increase of Bax, which was inhibited by GSPE. Moreover, GSPE restored the expression of Bcl-2 (Figures 5(a) and 5(b)), indicating that GSPE could decrease the Bax/Bcl-2 ratio. Furthermore, we used the A549 type II AEC cell line to investigate BLM-induced apoptosis of lung epithelial cells. Expectedly, the results of western blot indicated that after BLM treatment, the proapoptotic protein Bax was increased, whereas the level of the antiapoptotic protein Bcl-2 was decreased. In contrast, GSPE could reduce these protein levels and decrease the Bax/Bcl-2 ratio. These data suggest that GSPE could attenuate mitochondrial-associated cell apoptosis.

In this study, we showed that BLM administration induced increased protein level of TGF-*β*1 on day 21. The level of TGF-*β*1 was significantly lower in the BLM+GSPE treatment group compared with the BLM control group. This result suggested that GSPE could play an antifibrotic role via reducing TGF-*β*1. However, we could not confirm whether the potential anti-fibrotic effect of GSPE on BLM-induced pulmonary fibrosis animal models was due to the consequence of reduced oxidative stress or inflammation or the effect on fibrosis itself. Therefore, further studies would be needed to determine its contribution to Smad signaling or EMT of lung fibrosis. Besides, more mechanisms of GSPE on pulmonary fibrosis need to be explored so that GSPE can be used in clinical settings as a feasible approach to the treatment of IPF. Nevertheless, this study is the first to report the therapeutic effects of GSPE on BLM-mediated lung fibrosis and to elucidate the possible mechanism of GSPE in BLM-induced lung fibrosis.

In conclusion, we demonstrated that GSPE significantly ameliorated the histological changes and the level of collagen deposition in BLM-induced lungs. GSPE inhibited the BLM-induced IL-6 for early lung fibrosis and TGF-*β* for late lung fibrosis. In addition, GSPE significantly decreased the levels of ROS, and apoptosis was increased by BLM instillation in lung and epithelial cells. Furthermore, GSPE protected BLM-induced epithelial cell apoptosis via decreasing the Bax/Bcl2 ratio in vitro. Taken together, the findings demonstrate that GSPE can protect against BLM-induced pulmonary fibrosis in mice by inhibiting epithelial apoptosis through the inhibition of oxidative stress (Figure 5(c)), suggesting that GSPE may be useful in the treatment of IPF.

## Data Availability

The data used to support the findings of this study are included in the article.

## Abbreviations

GSPE: Grape seed proanthocyanidin extract
BLM: Bleomycin
IPF: Idiopathic pulmonary fibrosis
BAL: Bronchoalveolar lavage
AEC: Alveolar epithelial cell
IL-1ß: Interleukin-1ß
IL-6: Interleukin-6
ROS: Reactive oxygen species
TGF-*β*: Transforming growth factor beta 1
PARP-1: Poly [ADP-ribose] polymerase 1
Bax: BCL2 associated X
Bak: Bcl-2 homologous antagonist killer
Bcl-2: B-cell lymphoma 2.
